# Palladium catalyst imbedded in polymers of intrinsic microporosity for the Suzuki–Miyaura coupling reaction†

**DOI:** 10.1039/c8ra06214e

**Published:** 2018-10-16

**Authors:** Junwen Xu, Junjie Ou, Lianfang Chen, Haiyang Zhang, Shujuan Ma, Mingliang Ye

**Affiliations:** Key Laboratory of Separation Science for Analytical Chemistry, Dalian Institute of Chemical Physics, Chinese Academy of Sciences Dalian 116023 China junjieou@dicp.ac.cn mingliang@dicp.ac.cn; University of Chinese Academy of Sciences Beijing 100049 China

## Abstract

Polymers of intrinsic microporosity (PIMs) are porous polymers with rigid ladder-type chain structures. Synthesizing these polymers usually involves the step polymerization of two types of monomer, namely, active fluorine-substituted aromatic ring monomers and phenolic monomers. Herein, we report a new PIMs preparation method using self-synthesized fluorinated monomers and common monomer 5,5′,6,6′-tetrahydroxy-3,3,3′,3′-tetramethyl spirobisindane. The fluorinated monomers were synthesized through the imidization of tetrafluorophthalic anhydride and aromatic diamines. The resulting PIMs served as a support for palladium, with the formed catalyst showing potential for application in the Suzuki–Miyaura coupling reaction.

## Abstract 2

Polymers of intrinsic microporosity (PIMs) are porous polymers with rigid ladder-type chain structures.

## Graphical abstract

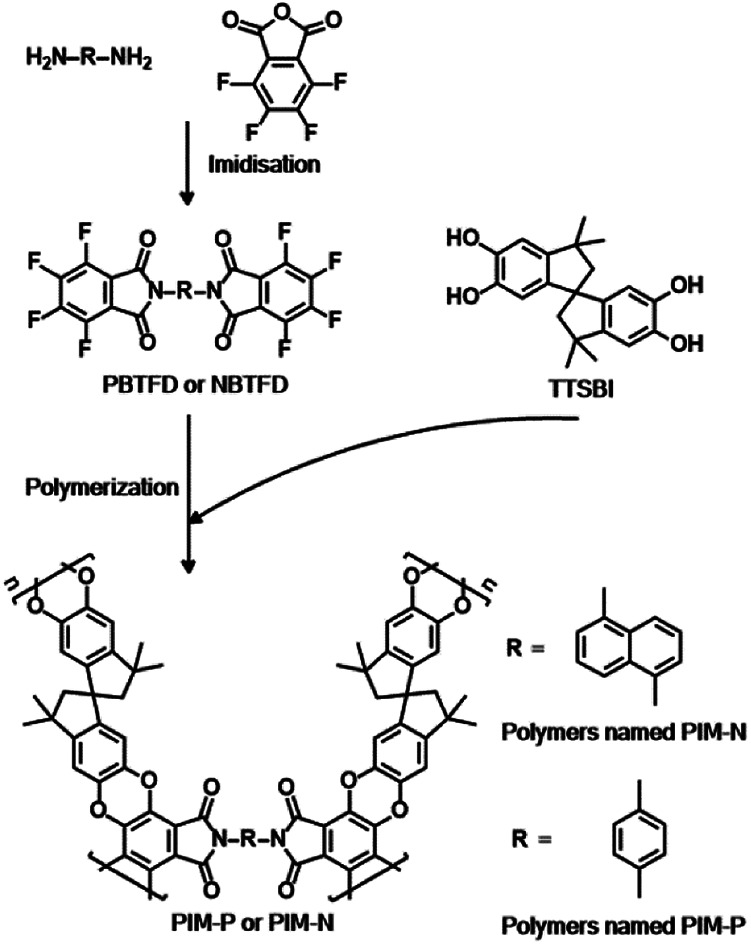

## Introduction

Polymers of intrinsic microporosity (PIMs) are a relatively new series of microporous polymers first reported by McKeown in 2004.^1,2^ The high Brunauer–Emmett–Teller (BET) surface area of PIMs originates from their unique rigid ladder-type main-chain structures with contorted centers against pore collapse and efficient space packing.^3,4^ This special structure relative to other microporous polymers means that the molecular chain of PIMs can be linear, which contributes to the fine solubility of some high-molecular-weight PIMs in organic solvents, such as dichloromethane, trichloromethane, and tetrahydrofuran. Solvent-soluble PIMs can be directly cast as microporous films with good gas permeability, allowing the separation of important gas pairs,^5–7^ CO_2_,^8–10^ H_2_,^11^ and other gases. In addition to gas separation, other PIM applications include gas storage,^12^ sensors,^13^ chiral separation,^14^ and energy conversion.^15^

The palladium-catalyzed Suzuki–Miyaura coupling reaction has played a significant role in the construction of biaryl compounds and substitution with aromatic moieties in organic synthesis, with applications in polymer synthesis and the modification of pharmaceuticals, natural products, and functional materials.^16,17^ Homogeneous catalysis of the Suzuki–Miyaura coupling reaction has been extensively investigated. However, difficulties in product separation and catalyst recycling have prevented wider industrial application. The immobilization of existing homogeneous palladium catalysts on a substrate is a potential solution to these problems. Numerous supports have been developed for palladium immobilization, including metal–organic frameworks (MOFs),^18^ covalent organic frameworks (COFs),^19^ hyper-crosslinked organic microporous polymers,^20^ active carbon,^21,22^ graphene,^23^ mesoporous silica,^24,25^ and zeolites.^26^ Supports with favorable effects must contain palladium binding sites and have high BET surface areas. These binding sites should effectively and powerfully bind palladium and sufficiently abundant to guarantee the binding effect and palladium quantity. ESI† with high BET surface areas can provide more contact sites for reactants and catalysts to enhance the reaction efficiency.

**Scheme 1:**
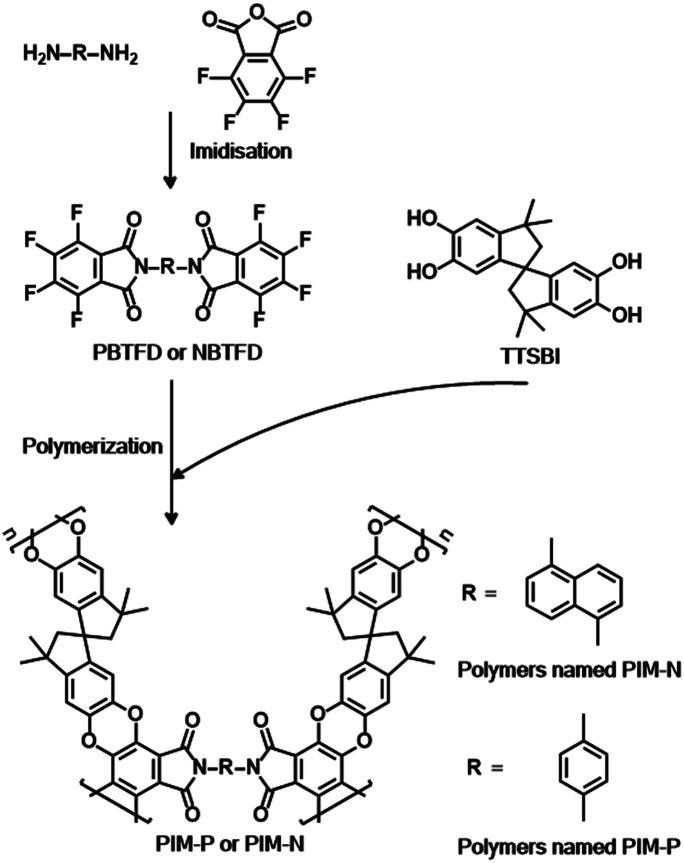
Synthesis of fluorinated monomers PBTFD and NBTFD, and construction of two PIMs (PIM-P and PIM-N).

Although PIMs have already been employed in catalysis,^27^ their application as catalyst supports is rare. PIMs possessing high BET surface areas and structures such as imides and cyano groups have been shown to be superior and versatile ligands for a variety of metal ions in coordination chemistry, meeting the requirements for supports. PIM-1 is a representative PIM derived from the step-growth polymerization of contortion donor 5,5′,6,6′-tetrahydroxy-3,3,3′,3′-tetramethyl-1,1′-spiro-bisindane (TTSBI) and linking fluorinated monomer 2,3,5,6-tetrafluoroterephthanlonitrile. Herein, we have designed a class of new PIMs with imide structures to serve as palladium supports using TTSBI as the contortion donor and two novel link monomers based on fluorinated phthalimide, namely 2,2′-(1,4-phenylene)bis(4,5,6,7-tetrafluoroisoindoline-1,3-dione) (PBTFD) and 2,2′-(naphthalene-1,5-diyl)bis(4,5,6,7-tetrafluoroisoindoline-1,3-dione) (NBTFD), respectively (Scheme 1). Their ^1^H NMR spectra are shown in Fig. S1 and S2 (ESI†). The imide structure in the linking monomer provides palladium binding sites. The palladium(ii)-coordinated PIMs material was conveniently synthesized by mixing PIMs with Pd(OAc)_2_ in dichloromethane at room temperature.

## Results and discussion

Phthalimide-based fluorinated monomers (PBTFD and NBTFD) were synthesized using a strategy similar to that reported by Makhseed *et al.*,^28^ wherein fluorinated monomers were synthesized by the one-step imidization reaction of tetrafluorophthalic anhydride with different monoamines in refluxing acetic acid. In our strategy, the aromatic diamines were used in the imidization reaction, resulting in the obtained polymers having a cross-linked structure, in contrast to the linear single-chain polymers reported previously. While these linear polymers were soluble in organic solvents such as chloroform, the polymers synthesized in this work were insoluble in almost all organic solvents due to their highly cross-linked structure.

**Fig. 1:**
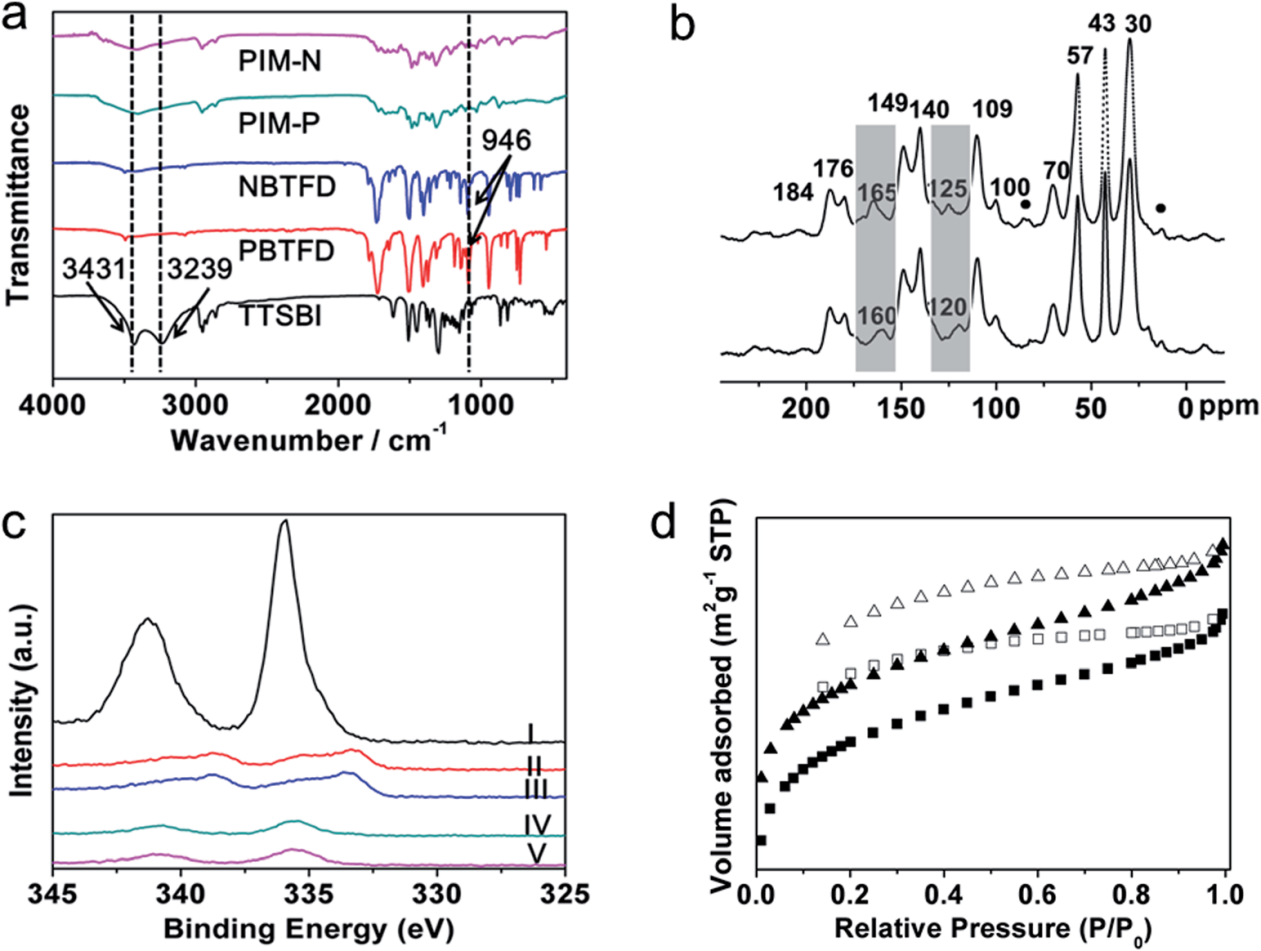
(a) FT-IR spectra of the three precursors and resulting PIMs; (b) ^13^C CP/MAS NMR spectra of polymers PIM-P (solid) and PIM-N (dots); (c) XPS spectra of (I) Pd(OAc)_2_, (II) Pd/PIM-N after one cycle, (III) Pd/PIM-P after one cycle, (IV) Pd/PIM-N, and (V) Pd/PIM-P; (d) nitrogen adsorption isotherms of PIM-P (square) and Pd/PIM-P (triangle).

Both PIM products and precursors were characterized by Fourier-transform infrared (FT-IR) spectroscopy, with the results presented in Fig. 1a. Characteristic bands at 2592, 2924, and 2861 cm^−1^ were attributed to C–H stretching vibrations of methyl and methylene groups in the spiral ring, which also appeared in the spectrum of monomer TTSBI. The bands were also found in the spectrum of the synthesized PIM because these groups did not participate in the polymerization. In the spectrum of monomer TTSBI, other bands at 3239 and 3431 cm^−1^ were attributed to O–H stretching vibrations. The bands were markedly weaker in the spectrum of the obtained PIMs because the phenol hydroxy groups were reactive sites in the polymerization. A strong C–F stretch at 946 cm^−1^ was observed in the spectra of monomers PBTFD and NBTFD. However, the C–F stretching vibration had disappeared in the spectra of PIM-P and PIM-N because these groups were also reactive sites in the polymerization. The results confirmed that fluorinated monomers PBTFD or NBTFD had polymerized with TTSBI. Pd/PIM-P and Pd/PIM-N were also characterized by X-ray photoelectron spectroscopy (Fig. S11†), which indicated residual C–F bonds were present in the polymers. However, the C–F content was too low to be detected using FT-IR. This result showed that very few of the C–F bonds had not participated in the polymerization.

The PIMs were next characterized using solid-state ^13^C cross-polarization magic angle spinning nuclear magnetic resonance (CP/MAS NMR) spectroscopy. As shown in Fig. 1b and S3c,† both PIM-P and PIM-N had four carbon signals with chemical shifts below 70 ppm. These peaks were located at 30, 43, 57, and ∼70 ppm and assigned to the carbon atoms in the spiral rings of the polymers. The spectra of PIM-P and corresponding catalyst Pd/PIM-P showed major differences, as depicted in Fig. 1b and S3d.† Firstly, the imide group carbonyl signal appeared at 160 ppm in the spectrum of PIM-P. However, this signal was shifted to 165 ppm in the spectrum of Pd/PIM-P. Secondly, the ^13^C signal for the N-substituted carbon on the aromatic ring was at 120 ppm in the PIM-P spectrum, but at 125 ppm in the Pd/PIM-P spectrum. To summarize, after binding palladium to the PIMs, the ^13^C chemical shift of the N-substituted carbon and imide carbonyl carbon were both shifted to a lower field by 5 ppm. This phenomenon resulted from electron donation from palladium to polymer imide groups, which confirmed the binding of Pd(OAc)_2_ with PIM-P. The same changes were also observed for PIM-N and Pd/PIM-N, indicating successful coordination of Pd with the polymers. In the ^13^C NMR spectra of PIMs and Pd/PIMs, peaks were observed at 170–190 ppm, which were attributed to carbon atoms from carboxyl groups and amide groups. These groups originated from the imide groups, indicating that some imide rings in the backbone were opened during polymerization. Peaks marked with round dots might originate from solvent residues. Other peaks in the spectrum were assigned to aromatic carbons in the polymer skeleton.

XPS spectroscopy was also employed to investigate the elemental compositions of the surface and coordination states of Pd species. As shown in Fig. S11,† XPS of Pd/PIM-P and Pd/PIM-N confirmed that C, O, N, F, and Pd elements were presented in the catalysts. The XPS spectra also showed the oxidation state of Pd species embedded in the PIMs. As shown in Fig. 1c, characteristic peaks of Pd^2+^ 3d_5/2_ and Pd^2+^ 3d_3/2_ appeared at 340.7 and 335.4 eV for Pd/PIM-P, and 340.7 and 335.7 eV for Pd/PIM-N, respectively. Compared with Pd(OAc)_2_ (341.3 and 336.0 eV), the binding energy was negatively shifted by around 0.5 eV, resulting from strong electron-donation from the imide groups in the PIM backbone. After employed the catalyst in the Suzuki–Miyaura coupling reaction, the Pd 3d binding energy region of Pd/PIM-P was deconvoluted into four peaks, with the two major peaks assigned to Pd(0), indicating that the bound palladium was almost completely reduced. The same was observed for Pd/PIM-N.

**Fig. 2:**
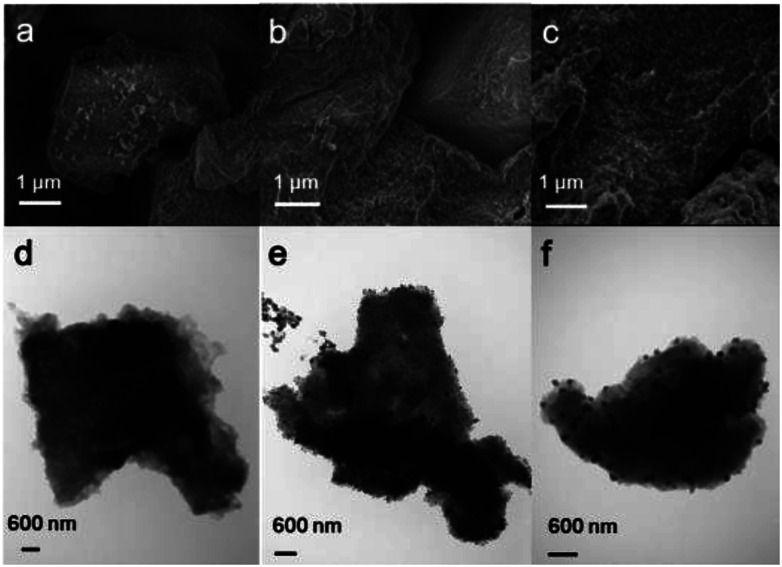
(a–c) SEM and (d–f) TEM images of (a and d) PIM-P, (b and e) Pd/PIM-P, and (c and f) Pd/PIM-P after one cycle.

The PIMs and Pd/PIMs were then investigated using scanning electron microscopy (SEM) and transmission electron microscopy (TEM). The SEM image (Fig. 2a) and TEM image (Fig. 2d) of PIM-P showed no differences. For catalyst Pd/PIM-P, evenly distributed dark dots were observed in the SEM image, although a similar state was not reflected in the TEM image. Comparing the TEM images of PIM-P and Pd/PIM-P, we concluded that palladium nanoparticles were present in the Pd/PIM-P catalyst. The SEM images of freshly synthesized Pd/PIM-P catalyst and used Pd/PIM-P catalyst were different, with bright dots observed on the used catalyst. This observation could indicate the agglomeration of Pd particles embedded in the polymers, resulting in deactivation of the used catalyst. Pd agglomeration was further proven by comparing the TEM image of the fresh catalyst with that of the used catalyst. As shown in Fig. 2e and f, the Pd particle size in the used catalyst was larger than that in the fresh catalyst. Furthermore, the uniformity of the particle distribution was inferior in the used catalyst. The same phenomenon was also observed for PIM-N and Pd/PIM-N, with the relevant images shown in Fig. S4–S9.†

The porous nature of PIM-P, PIM-N, Pd/PIM-P, and Pd/PIM-N was investigated using nitrogen adsorption–desorption analysis at 77 K. Fig. 1d and S10† show the nitrogen adsorption isotherms of the PIMs and PIM-based catalysts. Application of the BET model resulted in surface areas of 355, 322, 516, and 501 m^2^ g^−1^ for PIM-P, PIM-N, Pd/PIM-P, and Pd/PIM-N, respectively. The total pore volumes of PIM-P, PIM-N, Pd/PIM-P, and Pd/PIM-N were calculated as 0.27 (*P*/*P*_0_ = 0.99), 0.24 (*P*/*P*_0_ = 0.99), 0.31 (*P*/*P*_0_ = 0.99), and 0.31 cm^3^ g^−1^ (*P*/*P*_0_ = 0.99), respectively. According to these results, we concluded that both the BET surface areas and pore volumes showed an obvious increase after binding palladium to the PIMs. The synthesized PIMs were highly cross-linked under the reaction conditions. Therefore, palladium binding might help support the polymer skeleton, making the polymer chains less clustered and resulting in increased BET surface areas and pore volumes. Furthermore, the interpenetrating structure of PIMs was loosened when the polymers were immersed in dichloromethane during the palladium binding procedure. The palladium particles then entered the outstretched void. After the solvent was removed, the outstretched void could not shrink effectively, contributing to the increase in BET surface area. Data analysis showed that the pore sizes of the PIMs and Pd/PIMs were below 2 nm, categorized as micropores.

The catalytic activity of Pd/PIM in the Suzuki–Miyaura coupling reaction was investigated, with the results exhibited in Table 1. In a typical test run, phenyl bromide and phenylboronic acid were chosen as substrates, and a mixture of water and ethanol (equal volumes) was selected as the solvent. When 0.37 mol% of either catalyst (Pd/PIM-P and Pd/PIM-N) was applied in the reaction, the biphenyl yield was above 99% after 30 min at room temperature. Both catalysts showed good catalytic activity for the coupling reaction under mild conditions (ambient temperature). The coupling reactions did not proceed when PIMs without coordinated palladium were used as catalysts, demonstrating that bound palladium was responsible for the catalytic activity rather than the polymers. To examine the catalytic versatility, other substrates were also tested in the presence of Pd/PIM-N. As shown in Table S1,† the product yields in all reactions, except for that of substrates *o*-nitrophenylbromide and phenylboronic acid, were up to 93%. These results indicated that substituting the counterposition and interposition of bromobenzenes and phenylboronic acids slightly influenced the coupling reaction. However, *ortho*-substituted bromobenzenes, represented by *o*-nitrophenylbromide, *o*-tolylbromide, and *o*-tolylboronic acid, did not react well with phenylboronic acids under these reaction conditions owing to steric hindrance. The rate of the coupling reaction was also influenced by the amount of catalyst. When the palladium dose was 0.37 mol%, the all biphenyl yields were up to 93% after 30 min. When the dosage was increased four-fold, the time used to achieve a similar effect was decreased to 15 min. The catalyst recyclability was studied because it is vital for practical applications. However, the catalytic activity of Pd/PIMs seriously declined after one cycle. TEM, SEM, and XPS analysis were used to investigate the catalyst deactivation mechanism. After use, the supported palladium was reduced and palladium nanoparticles agglomerated, leading to catalyst deactivation.

**Table 1:** Catalytic activity of Pd/PIM-P in the Suzuki–Miyaura coupling reaction^a^

Entry	Aryl halide	Arylboronic acid	Time/h	Yield/%
1	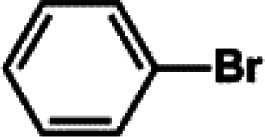	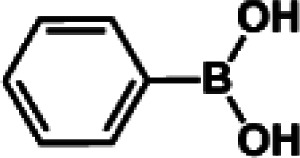	30	97
2	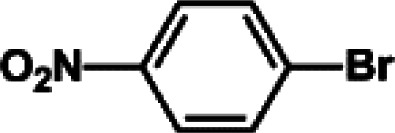	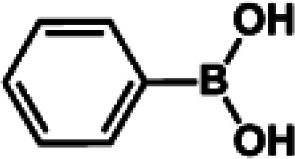	30	95
3	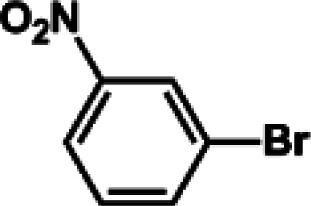	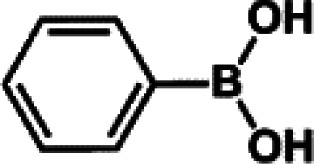	30	>99
4	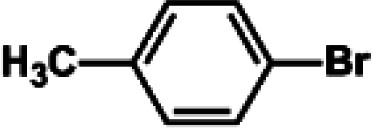	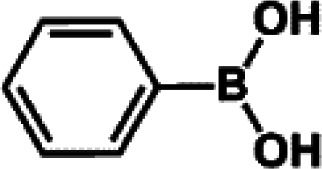	30	98
5	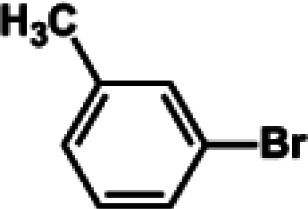	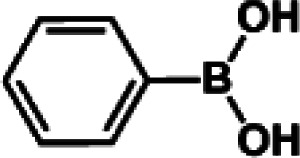	30	96
6	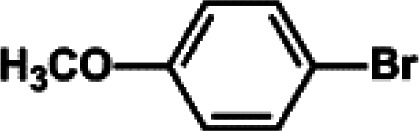	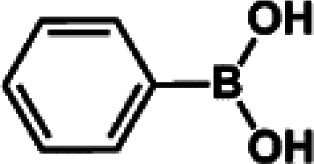	30	93
7	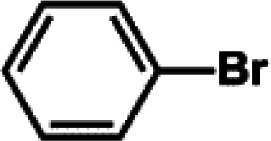	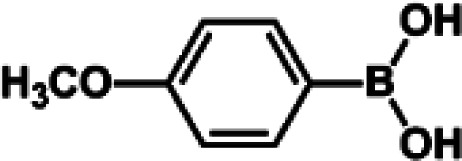	30	>99
8	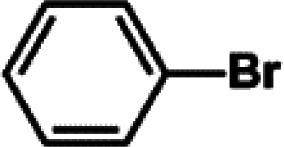	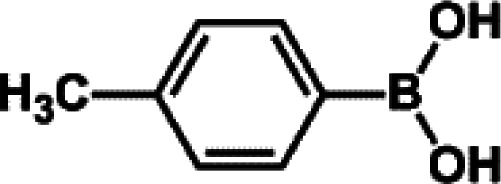	30	96
9	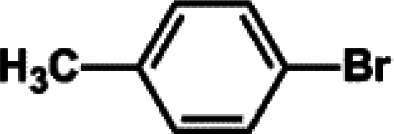	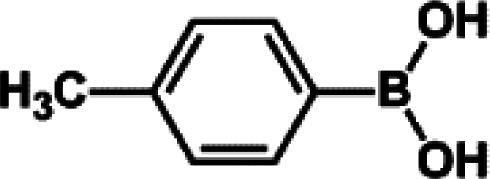	30	>99

a Reaction conditions: aryl bromide (1.0 mmol), phenyl boronic acid (1.5 mmol), alkali (3.0 mmol), EtOH/H_2_O (50 : 50, v/v, 4.0 mL), Pd/PIM-P (5.0 mg, 0.37 mol%), room temperature.

## Conclusion

In summary, we have successfully synthesized a new type of PIM with a cross-linked structure. The PIMs contained abundant imide groups that served as coordinating groups for metal ions. Binding palladium to the PIMs formed a catalyst with potential applications in the Suzuki–Miyaura coupling reaction. The catalytic activity was tested on bromobenzene and phenylboronic acid substrates, showing good performance in the first run. However, after one cycle, the catalyst exhibited a large loss in catalytic activity, which was attributed to reduction and agglomeration of the palladium catalyst nanoparticles. In this work, we have developed a new synthetic strategy for preparing PIMs that might be applied in other research fields.

## Experimental section

### Synthesis of monomer PBTFD

*p*-Phenylenediamine (0.108 g, 1.0 mmol) and 3,4,5,6-tetrafluorophthalic anhydride (0.484 g, 2.2 mmol) in a 50 mL flask were dissolved in glacial acetic acid (20 mL). The mixture was stirred at room temperature for 12 h and refluxed at 120 °C for 12 h. The precipitate was separated by centrifugation, washed with glacial acetic acid and acetonitrile successively three times, and dried at 80 °C under high vacuum for 12 h to yield a white powder (0.420 g, 82% yield). ^1^H NMR (500 MHz, DMSO-d_6_): *δ* 7.67 (s, 4H). MALDI-TOF/MS: calcd for [C_22_H_6_N_2_O_4_F_8_ + Na] 535.2552, [C_22_H_6_N_2_O_4_F_6_ + K] 551.3637; found 534.9682, 550.9340. FT-IR (powder, cm^−1^): 3493, 1783, 1727, 1646, 1510, 1406, 1373, 1314, 1089, 946, 749, 726, 543.

### Synthesis of monomer NBTFD

1,5-Diaminonaphthalene (0.158 g, 1.0 mmol) and 3,4,5,6-tetrafluorophthalic anhydride (0.484 g, 2.2 mmol) in a 50 mL flask were dissolved in glacial acetic acid (20 mL). The mixture was stirred at room temperature for 12 h and refluxed at 120 °C for 12 h. The precipitate was separated by centrifugation, washed with glacial acetic acid and acetonitrile successively three times, and dried at 80 °C under high vacuum for 12 h to yield a yellow powder (0.481 g, 86% yield). ^1^H NMR (500 MHz, DMSO-d_6_): *δ* 8.22 (d, 1H, *J* = 8.2 Hz), 7.87–7.74 (m, 2H). MALDI-TOF/MS: calcd for [C_26_H_6_N_2_O_4_F_8_ + Na] 585.3138, [C_26_H_6_N_2_O_4_F_6_ + K] 601.4224; found 584.9788, 600.9211. FT-IR (powder, cm^−1^): 3497, 1790, 1733, 1646, 1503, 1461, 1403, 1359, 1145, 1093, 946, 794, 752, 732.

### Synthesis of PIMs

To a stirred solution of monomer PBTFD (0.512 g, 0.5 mmol) and TTSBI (0.681 g, 2.0 mmol) in DMF (100 mL) was added K_2_CO_3_ (6.91 g, 50 mmol). The mixture was heated to 120 °C for 12 h. The reaction mixture was poured into excess deionized water to form a yellow precipitate that was separated by filtration and washed with dichloromethane in a Soxhlet extraction for 24 h to remove residues. The refined product was dried under high vacuum to yield PIM-P as a yellow powder (1.08 g, 97% yield). Anal. calcd for (C_32_H_24_NO_6_)_*n*_: C 74.12, H 4.67, N 2.70, O 18.51; found: C 69.28, H 5.79, N 2.81, O 13.82. FT-IR (powder, cm^−1^): 3407, 2954, 2863, 1718, 1670, 1515, 1486, 1450, 1384, 1313, 1031, 824, 757, 455.

The synthesis of PIMs using NBTFD as precursor used the same procedures, affording PIM-N as a brown powder (0.991 g, 85% yield). Anal. calcd for (C_34_H_25_NO_6_)_*n*_: C 75.13, H 4.64, N 2.58, O 17.66; found: C 69.86, H 5.87, N 2.56, O 13.49. FT-IR (powder, cm^−1^): 3419, 2954, 2863, 1722, 1669, 1487, 1451, 1316, 1214, 1031, 977, 874, 781.

### Synthesis of Pd/PIMs

To a stirred solution of Pd(OAc)_2_ (50 mg, 0.20 mmol) in dichloromethane (10 mL) was added Pd/PIM-P (0.20 g), and the mixture was stirred for 24 h. The resulting solid was separated by centrifugation, washed with acetonitrile three times, washed with dichloromethane in a Soxhlet extraction for 24 h, and then dried at 120 °C under high vacuum for 12 h to yield Pd/PIM-P as a brown powder (0.17 g, 74% yield). The Pd content was 7.8%, as determined by ICP.

The synthesis of Pd/PIM-N followed the same procedure, affording a brown powder (0.18 g, 80% yield). The Pd content was 8.2%, as determined by ICP.

### General procedure for the Suzuki–Miyaura coupling reaction

In a typical catalytic activity test of Pd/PIMs, aryl halide (1.0 mmol), phenylboronic acid (1.5 mmol), K_2_CO_3_ (3.0 mmol), and Pd/PIM-P (5.0 mg, 0.37 mol%) were added to water/ethanol (50 : 50, v/v, 4.0 mL). The reaction mixture was stirred at room temperature for 30 min. On completion, the reaction mixture was diluted with deionized water (20 mL) and extracted with ethyl acetate (10 mL) three times. The organic layers were combined and diluted to 100 mL with acetonitrile. The product yield was determined by HPLC using the following chromatography conditions: analysis column (packed with C18 AQ beads (5 μm, 12 nm)); column dimension, 0.46 cm ID × 16 cm; mobile phase, acetonitrile/H_2_O (60 : 40, v/v); flow rate, 1 mL min^−1^; detection wavelength, 218 nm.

## Conflicts of interest

There are no conflicts to declare.

## Supplementary Material

RA-008-C8RA06214E-s001
