# Supplementary material for: Palladium catalyst imbedded in polymers of intrinsic microporosity for the Suzuki–Miyaura coupling reaction
Source: RSC Adv. 2018 Oct 16;8(61):35205–10. doi: 10.1039/c8ra06214e (PMC9087636; doi:10.1039/c8ra06214e)
Supplement: RA-008-C8RA06214E-s001 [file RA-008-C8RA06214E-s001.pdf]

Electronic Supplementary Material (ESI) for RSC Advances.
This journal is © The Royal Society of Chemistry 2018

Supporting Information:

Palladium (Pd) catalyst imbedded in polymers of intrinsic microporosity for Suzuki-Miyaura coupling reaction

Junwen Xu,[†,‡] Junjie Ou,[*,†] Lianfang Chen,[†,‡] Haiyang Zhang,[†] Shujuan Ma,[†] Mingliang Ye[*,†]

[†]Key Laboratory of Separation Science for Analytical Chemistry, Dalian Institute of Chemical Physics, Chinese Academy of Sciences, Dalian 116023, China

[‡]University of Chinese Academy of Sciences, Beijing 100049, China

Corresponding Author

[*]E-mail: junjieou@dicp.ac.cn (J. Ou)

[*]E-mail: mingliang@dicp.ac.cn (M. Ye)

Table of Contents

1. Chemicals and Instruments

2. Supplementary Figures and Tables

Figure S1. ^{1}H NMR spectra of monomer NBTFD.

Figure S2. ^{1}H NMR spectra of monomer PBTFD.

Figure S3. ^{13}C CP/MAS NMR spectra of PIM-N (c) and catalysts Pd/PIM-N (d).

Figure S4. SEM image of PIM-N.

Figure S5. SEM image of Pd/PIM-N.

Figure S6. SEM image of Pd/PIM-N after one cycle.

Figure S7. TEM image of PIM-N.

Figure S8. TEM image of Pd/PIM-N.

Figure S9. TEM image of Pd/PIM-N after one cycle.

Figure S10. Nitrogen adsorption isotherms PIM-N (circular) and Pd/PIM-N (rhombus).

Figure S11. The XPS image of Pd/PIM-P and Pd/PIM-N.

1. Chemicals and Instruments

p-Phenylenediamine, 1,5-naphthalenediamine, tetrafluorophthalic anhydride 5,5',6,6'-tetrahydroxy-3,3,3',3'-tetramethyl spirobisindane were purchased from TCI chemical (Shanghai, China). Palladium (II) acetate and anhydrous DMF was purchased from Sigma-Aldrich (St Louis, Mo, USA). Dichloromethane, glacial acetic acid, ethanol, acetonitrile and potassium carbonate anhydrous (K_2CO_3) were obtained from Kemiou Chemical Regent Co., Ltd. (Tianjin, China). The water was doubly distilled and purified by Milli-Q system (Millipore Inc., Milford, MA, USA). The C18 AQ beads (5 μm, 120 Å) were obtained from MichromBioResources (Auburn, CA).

FT-IR spectra were obtained on a spectrometer with KBr pellets containing polymer sample (Thermo Scientific, USA). Elemental analysis was carried out on EMIA-8100H and EMG-930 (HORIBA, Japan). Solid-state ^{13}C cross-polarization magnetic angle spinning (CP-MAS) NMR spectra and ^{1}H NMR spectra were recorded on a Bruker Advance III 500 NMR spectrometer (Germany). X-ray photoelectron spectroscopy (XPS) data were acquired using an ESCALAB 250Xi XPS spectrometer with an Al Kα X-ray source (Thermo Scientific, USA). The morphology study of porous organic polymer was carried out on a scanning electron microscopy (SEM, Gemini SEM 300, Zeiss, Germany). Transmission electron microscopy (TEM) was conducted on a JEOL 2000 EX electronic microscope (Japan) with an accelerating voltage of 120 keV. Nitrogen adsorption-desorption measurements were performed on a Micromeritics ASAP 2460 (USA) surface area analyzer and pore size analyzer. The samples were outgassed under vacuum for 7 h at 120 ^{o}C before measurements. The surface area was calculated via the Brunauer-Emmett-Teller (BET) method. Chromatography analysis was performed on UPLC (Water, USA).

2. Supplementary figures and tables

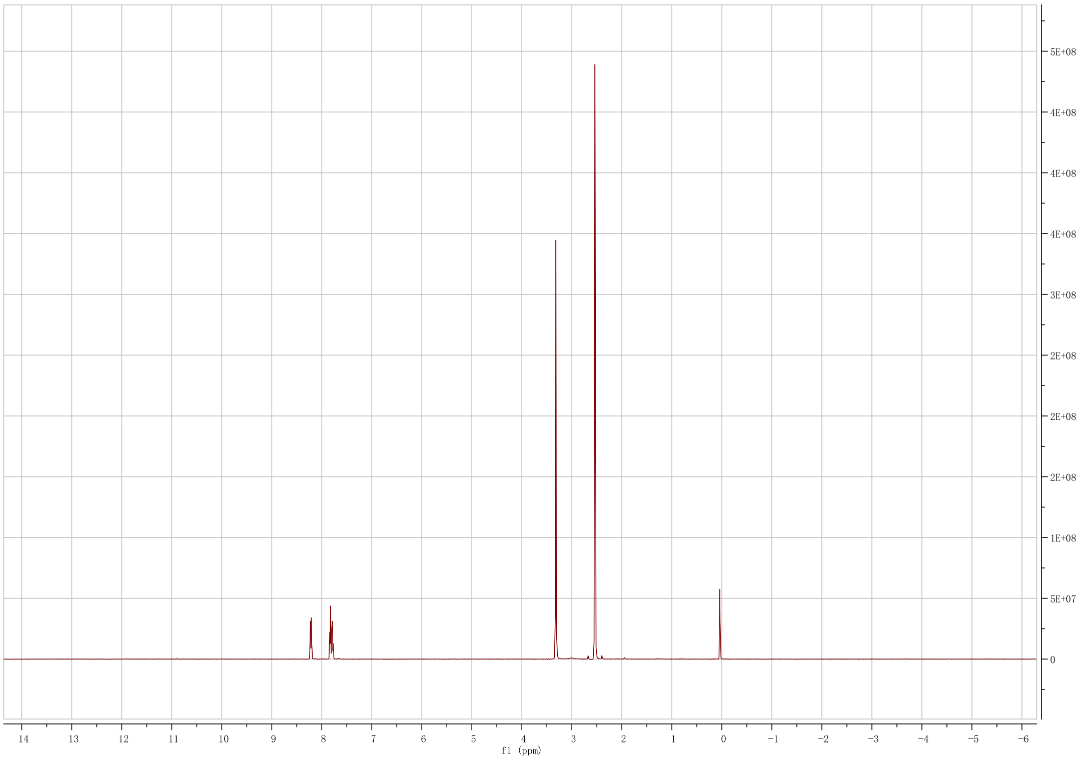

Figure S1. ^{1}H NMR spectra of monomer NBTFD.

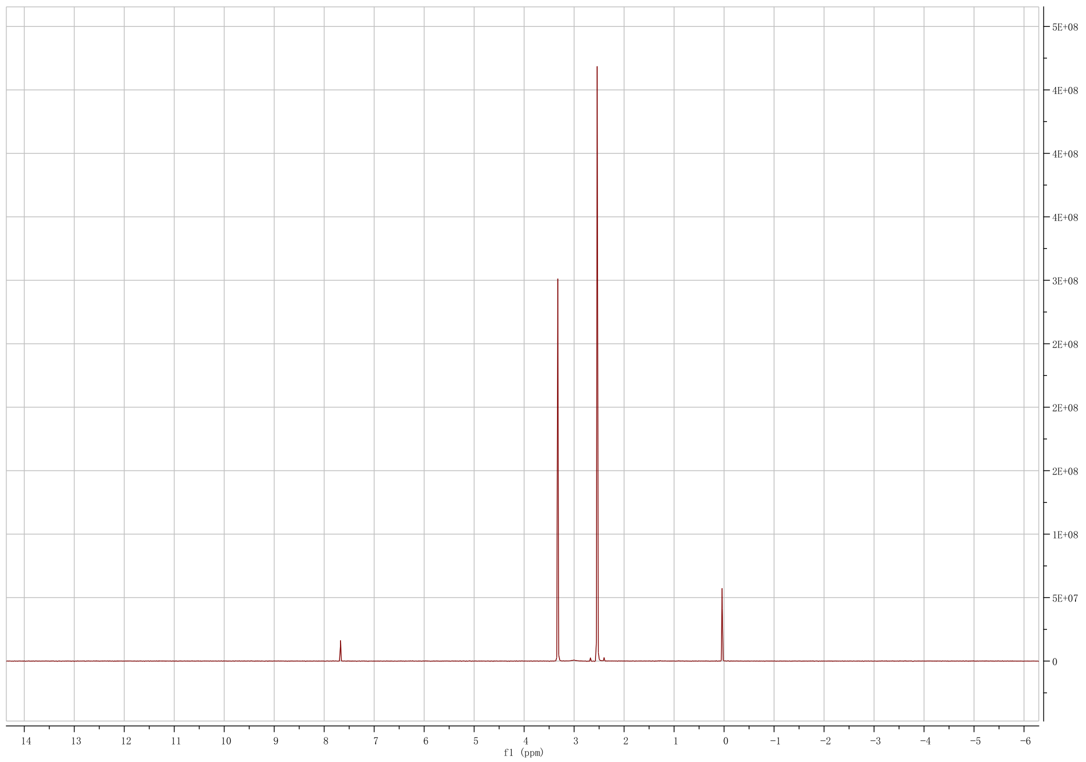

Figure S2. ^{1}H NMR spectra of monomer PBTFD.

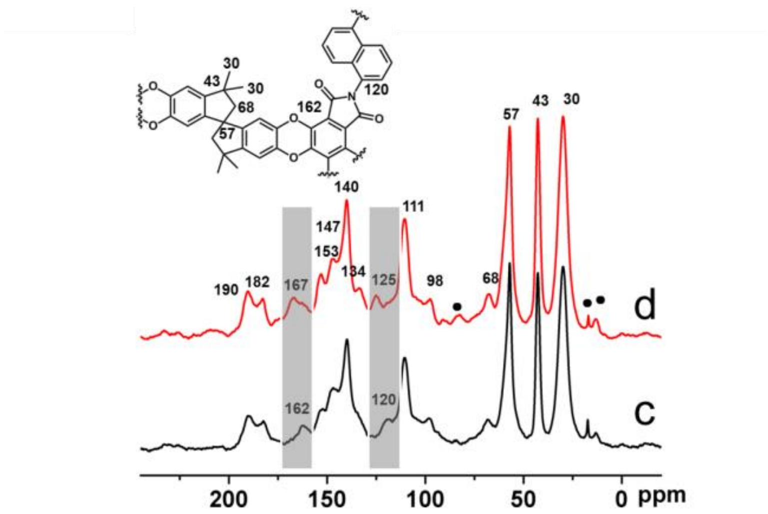

Figure S3. ^{13}C CP/MAS NMR spectra of PIM-N (c) and catalysts Pd/PIM-N (d).

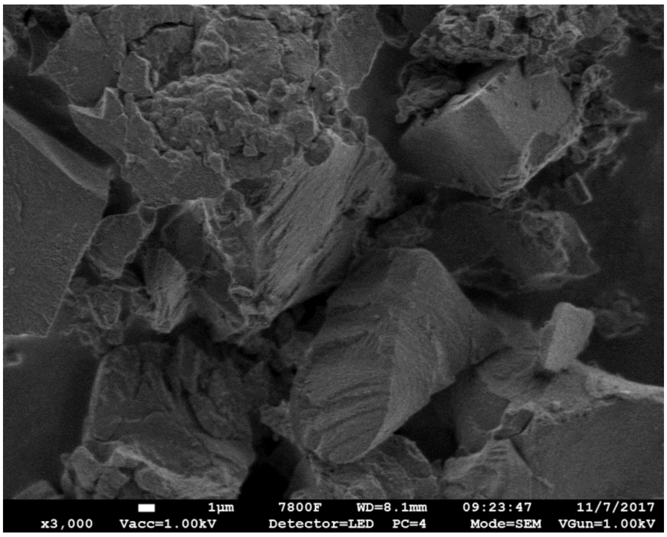

Figure S4. SEM image of PIM-N.

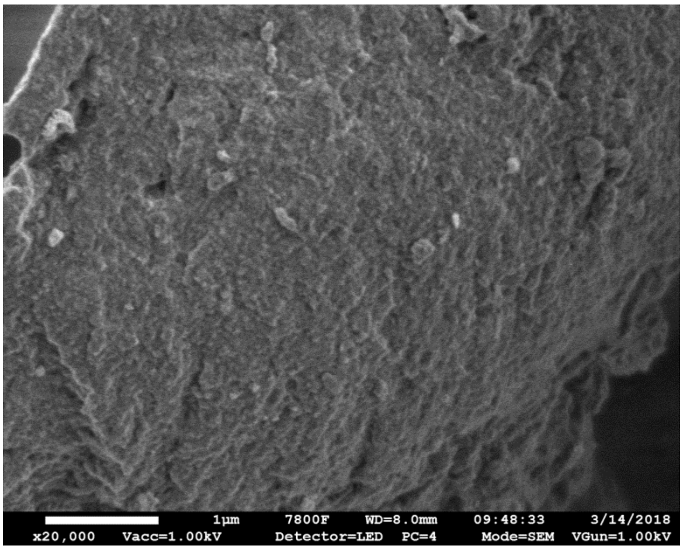

Figure S5. SEM image of Pd/PIM-N.

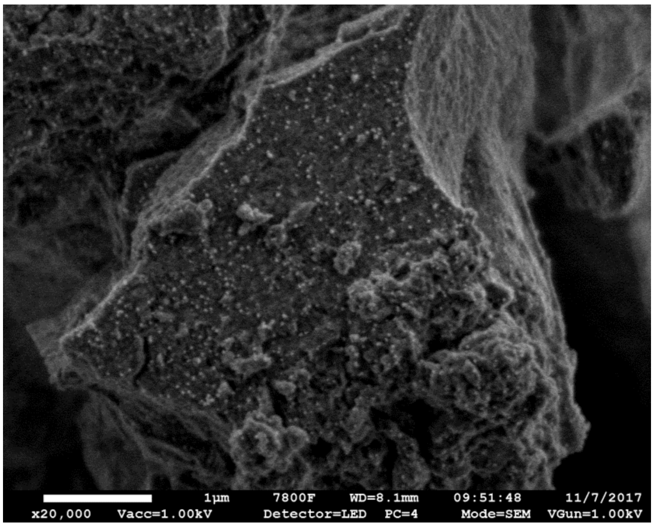

Figure S6. SEM image of Pd/PIM-N after one cycle.

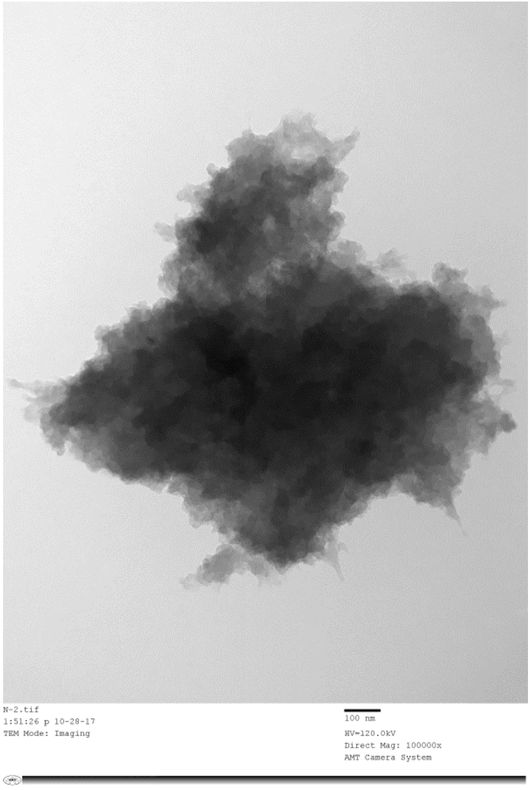

Figure S7. TEM image of PIM-N.

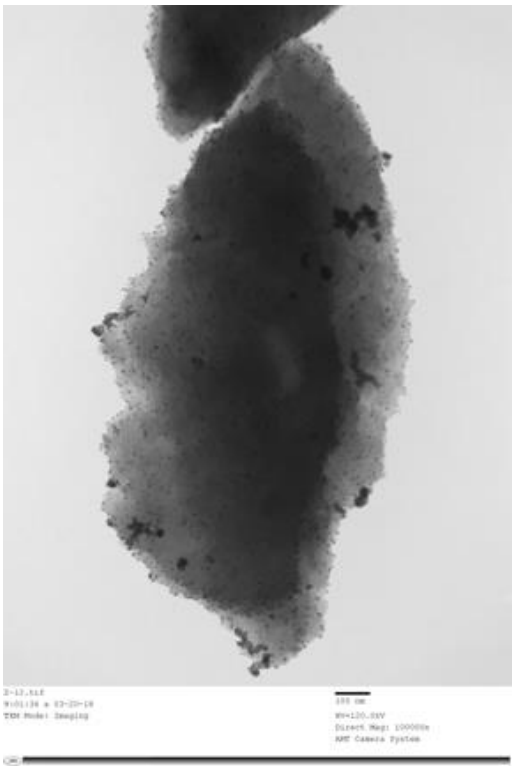

Figure S8. TEM image of Pd/PIM-N.

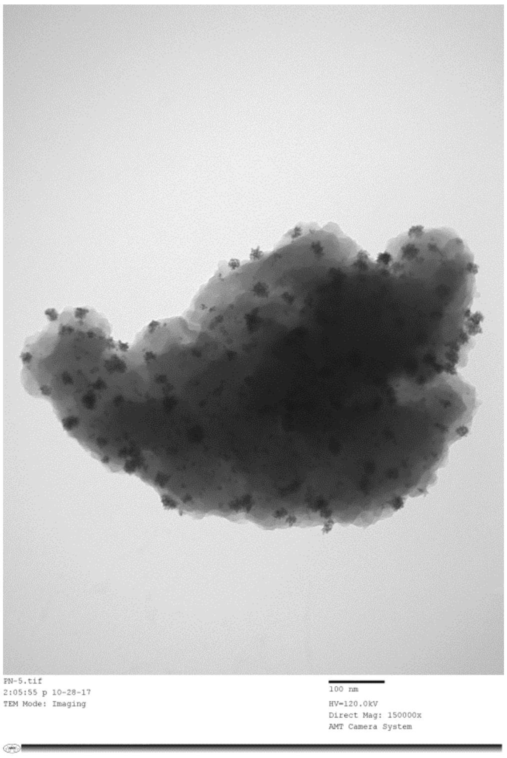

Figure S9. TEM image of Pd/PIM-N after one cycle.

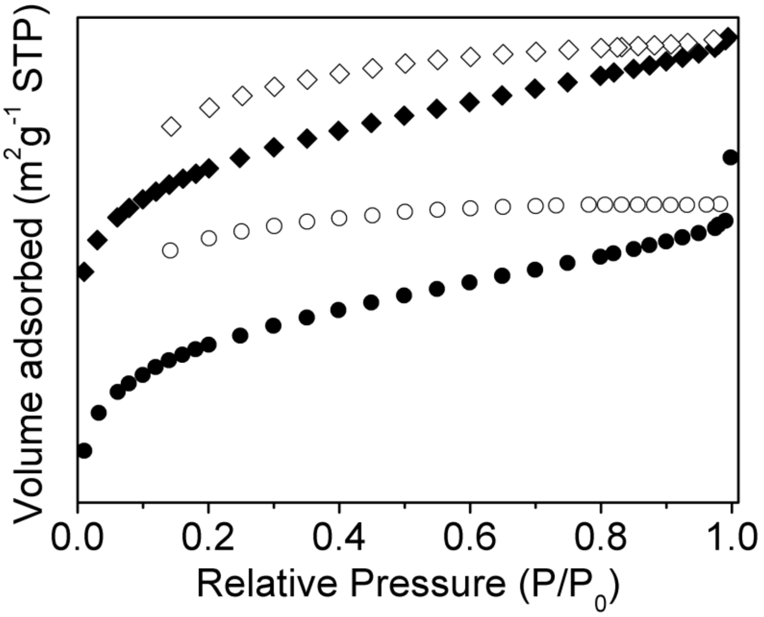

Figure S10. Nitrogen adsorption isotherms PIM-N (circular) and Pd/PIM-N (rhombus).

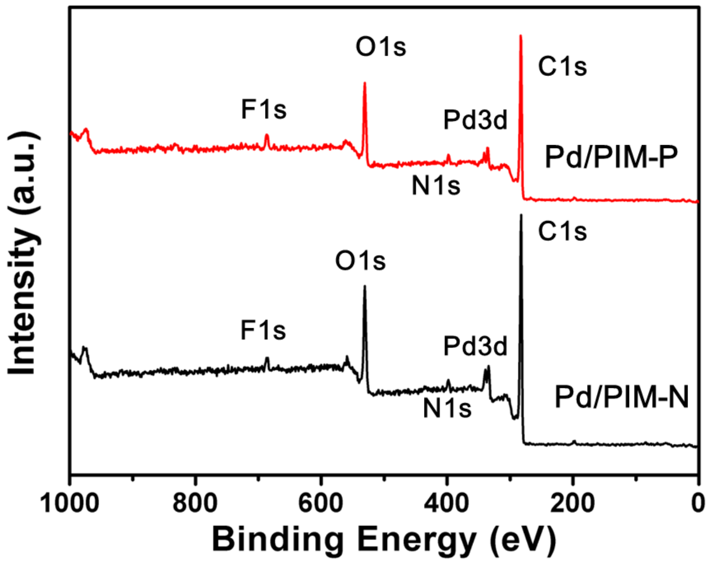

Figure S11. The XPS image of Pd/PIM-P and Pd/PIM-N.
